# Comparative analysis of A-to-I editing in human and non-human primate brains reveals conserved patterns and context-dependent regulation of RNA editing

**DOI:** 10.1186/s13041-017-0291-1

**Published:** 2017-04-06

**Authors:** Richard T. O’Neil, Xiaojing Wang, Michael V. Morabito, Ronald B. Emeson

**Affiliations:** 1 Vanderbilt Brain Institute and Division of Nephrology, Vanderbilt University School of Medicine, Nashville, TN USA; 2 Department of Biomedical Informatics, Vanderbilt University, Nashville, TN USA; 3 Department of Pharmacology, Vanderbilt University, Nashville, TN USA; 4 Departments of Pharmacology, Molecular Physiology and Biophysics, and the Vanderbilt Brain Institute, Vanderbilt University, Nashville, TN USA

## Abstract

A-to-I RNA editing is an important process for generating molecular diversity in the brain through modification of transcripts encoding several proteins important for neuronal signaling. We investigated the relationships between the extent of editing at multiple substrate transcripts (5HT2C, MGLUR4, CADPS, GLUR2, GLUR4, and GABRA3) in brain tissue obtained from adult humans and rhesus macaques. Several patterns emerged from these studies revealing conservation of editing across primate species. Additionally, variability in the human population allows us to make novel inferences about the co-regulation of editing at different editing sites and even across different brain regions.

**Electronic supplementary material:**

The online version of this article (doi:10.1186/s13041-017-0291-1) contains supplementary material, which is available to authorized users.

## Introduction

Modification of messenger RNAs by adenosine-to-inosine (A-to-I) editing is frequently observed in metazoan transcripts and represents a potentially important mechanism for facilitating advantageous molecular diversification in mammals. This kind of RNA editing has been postulated to play a prominent role in nervous system function by re-coding transcripts such that they encode functionally distinct proteins. A-to-I editing involves targeted conversion of specific adenosines on substrate transcripts to inosines by hydrolytic deamination, a reaction carried out by a family of enzymes known as Adenosine Deaminases Acting on RNA (ADARs) which interact with target substrates by binding regions of double stranded RNA. A-to-I editing effects RNA function because inosine base pairs with cytosine with similar Watson-Crick geometry as guanosine. As such, it behaves like guanosine in most cellular contexts including translation, alternative splicing, and RNA induced gene silencing [1] [2]. Importantly, alterations in protein function as a result of editing have been observed for several neurotransmitter receptors ranging from alterations in intercellular signaling for 5HT_2C_ receptors [3] [4]to changes in biophysical properties of ion channel subunits GLUR2 [5], GLUR4 [6], and GABRA3 [7]. While A-to-I editing is recognized as an important and ubiquitous phenomenon in mammals, little is known about how the process is regulated at individual sites and in distinct tissues. Knockout studies in mice [8] [9] [10] [11] indicate that the two ADAR enzymes (ADAR1 and ADAR2) have overlapping substrate specificities in certain cases and more distinct specificities in others. Previous work has indicated that many of the sites examined in this study are edited by both Adar1 and Adar2 in mice (5HT2C A site, B site, and C sites; GLUR4-Flip/Flop R/G sites, CADPS, and Gabra3), while the GLUR2 Q/R site and the D site on the 5HT2C receptor appear to be primarily targeted by Adar2. Conversely, the substrate specificity of ADAR enzymes at MGLUR4 While these studies do not clearly delineate ADAR substrate specificity in all relevant contexts they suggest that the unique mechanisms regulate A-to-I editing at each respective substrate. The following analyses were carried out to test the hypothesis that A-to-I editing is regulated by conserved substrate-specific and brain region-specific mechanisms. We focused on several A-to-I editing events in open reading frames which alter amino acid codons in transcripts important for synaptic communication in the CNS. This was accomplished by performing ‘head-to-head’ comparisons of A-to-I editing profiles in brain tissue samples harvested from striatum and cortex of both human and *rhesus macaque* looking specifically for correlations in the extent of editing across and between substrates as well as with ADAR mRNA levels.

For this study, we simultaneously quantify the extent of editing at several substrates in cortical and striatal tissue samples by targeted multiplex transcript analysis [12]. Interestingly, we observed similar patterns of editing for many of these substrates in human and *rhesus macaque* brains supporting the notions that the precise extent of editing at each of these substrates is conserved, tightly regulated, and functionally important. These results provide evidence that editing at certain substrates may be co-regulated within certain anatomical contexts and regulated by divergent mechanisms in others. When comparing ADAR mRNA expression levels with editing profiles we observe no direct relationship between mRNA expression and the extent of editing at any site analyzed in this study, supporting the hypothesis that A-to-I editing is regulated downstream of ADAR expression. Interestingly, we did observe a direct relationship between the mRNA expression levels of ADAR1 and ADAR2 mRNA transcripts over a large dynamic range in both species. Furthermore, observations in the human population indicate that certain individuals harbor multi-substrate deficiencies in A-to-I editing that generalize across each of the analyzed substrates while others display apparent deficiencies in editing at only a few substrates in certain neuroanatomical contexts.

## Results

### Covariant analysis of A-to-I editing

It should be noted that the human tissue samples represent a semi-matched cohort originally obtained to test the hypothesis that RNA processing of 5HT2C transcripts was aberrant in individuals with a genetic disorder known as Prader-Willi Syndrome (PWS). After observing that 5HT2C editing was not altered in the PWS we decided to leverage the remaining available tissue to investigate other aspects of the regulation of RNA editing presented in this study. After we analyzed editing in each tissue sample, we first verified that PWS diagnosis, sex, age, and postmortem interval (PMI) did not have an effect on patterns of editing or RNA expression. Correlation analysis (ANCOVA *p* > .05) did not reveal any evidence that the parameters discussed here are significantly influenced by diagnosis, sex, age or PMI (Additional file 1: Figure S1).

### Overall patterns of editing in the brain

**Fig. 1:**
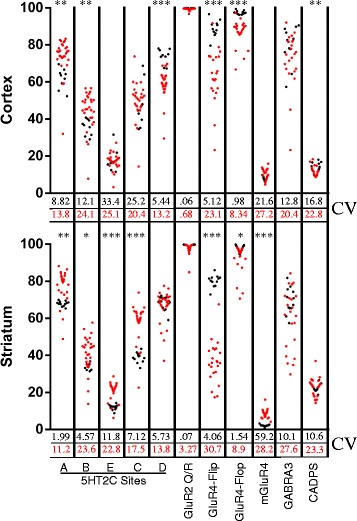
A-to-I editing in brain tissue from individual humans and *rhesus macaques*. Each *red dot* represents data from a single human sample and each *black dot* represents data from a *rhesus macaque* sample. The extent of editing is indicated on the Y-axis and the numbers below each column represent coefficient of variation for each species represented in the corresponding color. t-tests were used to compare the differences in the mean extent editing between the two species. (*p < .01 **p < .001 ***p < .0001)

The extent of RNA editing at 11 different sites within 6 substrate transcripts (GLUR2, GLUR4, GABRA3, CADPS, MGLUR4, and 5HT2C) were analyzed by multiplexed targeted transcript analysis which takes advantage of massively parallel sequencing on the MiSeq sequencing platform (Illumina, San Diego CA). RNA editing was detected at each site in all brain samples from humans and monkeys. The mean extent of editing observed for each respective editing site in these two species were generally similar, however considerably more variability was observed in the human population compared to the monkey at each substrate (Fig. 1). Importantly, the variability in the human population was largely driven by a few individuals who displayed profound deficiencies in editing at a few, or in some cases all, of the substrates analyzed. In both species, the highest levels of variability in editing were observed at transcripts encoding GABRA3. This relatively large variability was observed in both brain regions of these species and could indicate active dynamic regulation of editing at GABRA3 encoding transcripts.

While the mean extent of editing at most sites was found to be similar in monkey and human, the extent of editing at GLUR4-Flip and GLUR4-Flop transcripts is higher in both brain regions in the monkey (Cortex: Flip +25 *p* < .0001, Flop +11 *p* < .0001; Striatum: Flip +44 *p* < .0001, Flop +6 *p* < .01,). Additional interspecies differences were observed for the extent of editing at various substrates in specific brain regions, these are summarized in Fig. 1. Divergence in the extent of editing at certain substrates while other substrates retain similar patterns across species supports the hypothesis that dynamic regulation of editing occurs in a substrate specific rather than universal manner [13].

**Fig. 2:**
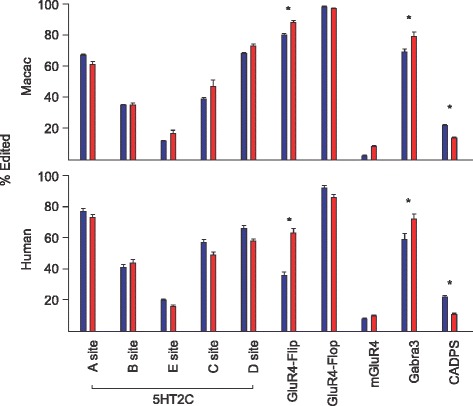
Comparison of the average extent of editing in striatum and cortex. The extent of editing at each site is compared between striatum (*blue*) and cortex (*red*) revealing several conserved patterns spatial regulation of editing in primate brains. T-tests were used to compare means of the two brain regions, asterisks are only plotted when similar results are observed in both species and represent the *p*-value obtained comparing means of each brain region using T-tests (*p < .01)

The patterns of editing across different anatomical contexts appear to be highly conserved in monkeys and in humans. The extent of GLUR4-Flip and GABRA3 editing is significantly higher in the cortex then the striatum, irrespective of species (*p* < .01). CADPS editing was significantly lower in the cortex than in striatum in both species (*p* < .01) (Fig. 2). The conserved spatial dynamics of A-to-I editing in these two species supports the idea that precisely regulating the editing profiles of certain transcripts is vitally important for normal CNS function.

### Co-regulation of RNA editing

When profiling the extent of editing in the human cohort we noticed higher levels of variability than we typically observe in rodent cohorts [12]. This was largely driven by a few individuals with editing deficiencies in editing at specific substrates, or in some cases deficiency in editing at all substrates. We took advantage of these deviations to examine the possibility that editing efficiency at certain substrates may be linked with each other, or if deficiencies in editing manifest across multiple brain regions.

**Fig. 3:**
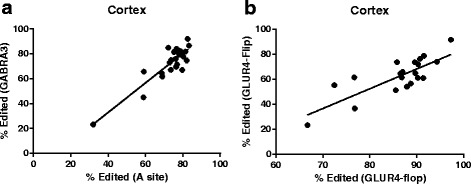
The extent of editing at each substrate was compared to all other substrates within each brain region by linear regression analysis. Relationships resulting in a *p* < .01 are shown. **a** Correlation of 5HT2C A site and GABRA3 editing in the cortex (*p* < .0001 *r* ^2^ = .8) **b** Correlation of GLUR4-Flip and GLUR4-Flop editing in the cortex. (*p* < .0001 *r* ^2^ = .62)

Our observations support the previously reported model that editing at each of the five sites on 5HT2C transcripts are co-regulated [14]. The extent of editing at each site predicts the relative extent of editing at each of the other five editing sites indicating that a single rate limiting step plays a pivotal deterministic role controlling editing at each of the 5HT2C editing sites. This finding is not surprising considering that each of these sites is located in close proximity on the same substrate transcript. However, we were surprised to observe a direct correlation between the extent of editing at 5HT2C transcripts and GABRA3 encoding transcripts in the cortex (Pearson correlation *p* < .0001, r^2^ = .8, Fig. 3b). This indicates a predictive relationship between the extent of editing at any one of the 5HT2C editing sites and that of the GABRA3 editing site. However, this relationship was not observed in striatum. This result leads us to speculate the hypothesis that the mechanisms or cell types regulating editing at these two substrates are similar in cortex and distinct in striatum.

We also observed direct correlation in the extent of editing at GLUR4-Flip and GLUR4-Flop transcripts in the cortex (Pearson correlation *p* > .001 r^2^ = .62). Thus, the extent of editing at GLUR4-Flip is a strong predictor of the extent of editing at GLUR4-Flop. This direct relationship could be related to the fact that these transcripts are generated by alternative splicing of the same pre-mRNA [15], and thus are subject to similar regulatory mechanisms. This effect was not observed in striatum further supporting a model where context-specific mechanisms play a role in regulating A-to-I RNA editing in the brain.

### Similar regulation of editing across brain regions

**Fig. 4:**
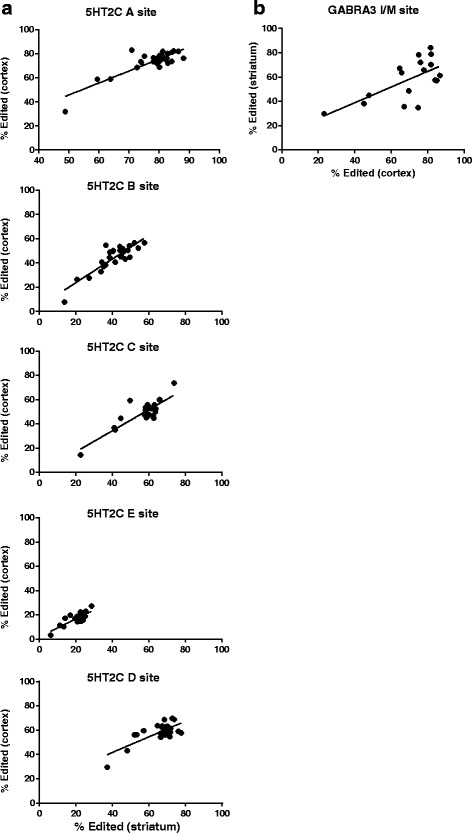
Correlation of editing between the striatum and cortex. Relationships resulting in a *p* < .01 are shown. **a** Linear regression analysis of 5HT2C editing in the frontal cortex and striatum (all sites; *p* < .0001, A site *r* ^2^ = .71, B site *r* ^2^ = .76, E site *r* ^2^ = .72, C site *r* ^2^ = .64, D site *r* ^2^ = .56). **b** Linear regression analysis of GABRA3 editing in the striatum and cortex (*p* = .005, *r* ^2^ = .42)

The inter-individual variability also allowed us to address the hypothesis that editing at some substrates could be regulated similarly across brain regions. We tested this by asking if editing in one brain region might accurately predict the extent of editing at that same transcript in another brain region of each individual. The extent of editing at 5HT2C receptor transcripts is positively correlated between cortex and striatum (Pearson correlation *p* < .0001 r^2^ > .5 for each site Fig. 4a). Similarly, we also observed evidence for co-regulation of editing at GABRA3 transcripts in cortex and striatum (*p* = .005 r^2^ = .42) albeit to a lesser extent than observed for 5HT2C editing. These results further support the hypothesis that editing at 5HT2C transcripts and GABRA3 transcripts may be regulated similarly in these two brain regions.

GLUR2 also displays a high level of correlation between brain regions but these results are difficult to interpret due to the limited range; as ~98-99% of the transcripts were edited in all tissues from all individuals with the exception of one patient. Interestingly, this individual had the lowest levels of GLUR2 Q/R site editing in both striatum and cortex implying some generalized deficiency in editing at this particular substrate.

Attempts to correlate editing across brain regions and substrates in the *rhesus macaque* cohort were precluded by the relatively low levels of inter-animal variability. Interestingly, despite the expected genetic diversity of this population, the extent of editing varied little between individuals (Fig. 1). These animals lived in highly controlled environments compared to the real-world experienced by the individuals in the human cohort. This observation supports the notion that some environmental factors may influence the extent of editing, although we cannot rule out the contribution of significant genetic variation within the human cohort.

### Adar Expression

**Fig. 5:**
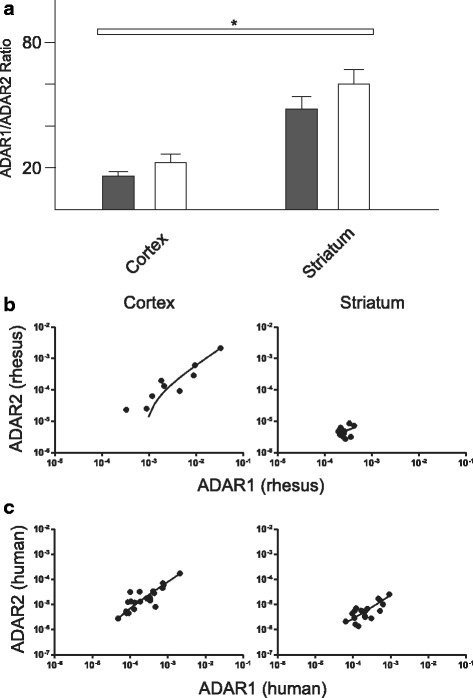
ADAR1 and ADAR2 expression in human and *rhesus macaque* brain tissue. **a** The mean ADAR1:ADAR2 ratio in each brain region is plotted (human results indicated by *shaded bars*) and no significant differences were observed between species. *Asterisks* represent significant differences between mean combined ratios of both species in each brain region as determined by *T*-test (*p < .01, **p < .001). **b** Linear regression analysis of ADAR1 and ADAR2 expression in *rhesus macaque* reveals a significant correlation between ADAR expression in cortex (*p* < .0001, *r* ^2^ = .93) **c** Linear regression analysis of ADAR1 and ADAR2 expression in human reveals a significant correlation between ADAR expression in cortex (*p* < .0001, *r* ^2^ = .91) and striatum (*p* < .0001, *r* ^2^ = .72)

We investigated the relationship between ADAR mRNA expression and A-to-I editing by comparing overall mRNA expression levels with the editing profiles determined for each sample. In primates, ADAR1 and ADAR2 are located on Chromosome 1 and Chromosome 21 respectively and the pre-mRNAs encoded by each undergo alternative splicing generating several unique transcripts [16–18]. The Taqman Probe (Life, CA) assays used for these studies detect all major ADAR1 and ADAR2 variants respectively. The probes were designed to detect all splice variants of each respective ADAR so the data presented represents a sum total of ADAR mRNA expression. ADAR1 mRNA was found to be 20–60 fold more abundant than ADAR2 in each tissue analyzed. Interestingly, we found that each respective tissue had a characteristic ratio of ADAR1:ADAR2 mRNA and that this ratio was maintained over large expression range (Fig. 5; Cortex ADAR1:ADAR2 18.64+/−2.09, Striatum ADAR1:ADAR2 52.46 +/−4.7). These brain region specific characteristic mRNA ratios were similar in both primate species (Fig. 3; *t*-test Human vs Monkey mRNA ratio *p* > .05 in each brain region). However, neither the ADAR mRNA expression levels nor the ADAR1:ADAR2 ratios correlate with the extent of editing at any of the substrates analyzed in this study (Additional file 2: Figure S2 and Additional file 3: Figure S3) (). The consistent ratio of ADAR1:ADAR2 across this large range supports the hypothesis that transcription of these two genes may be co-regulated and suggests that the ratio of ADAR1:ADAR2 is more important for normal physiology than the absolute level of expression.

## Discussion

Our analysis of A-to-I editing in dissected brain regions from humans and non-human primates allowed us to make several observations and revealed some patterns that may be useful for understanding the regulation of A-to-I editing. One of the most interesting observations from the human cohort was the remarkable inter-individual variability. This contrasts with observations made in rodents and primates reared in a laboratory setting which display more similar editing profiles [12] [19]. We decided to leverage the inter-individual variability in editing within this human cohort to investigate inter-substrate and cross- anatomical regulation of A-to-I editing. This revealed that editing at certain sites might predict the extent of editing at other sites as observed for GABRA3 and 5HT2C. It also revealed that the extent of editing at 5HT2C transcripts generalizes across at least two distinct brain regions analyzed in these studies. Many of the individuals in this cohort expressed ‘normal’ editing profiles for many substrates while displaying deficiencies in editing at only a few sites.The fact that these deficiencies were generally not observed in more than one brain region in most subjects suggests that the deficit does not result from an inherent polymorphism at the substrate transcript effecting secondary structure as such a polymorphism would be expected to manifest across different anatomical contexts. The individuals with low editing at certain substrates in discrete brain regions could represent examples of region specific plasticity in editing efficiency, however we cannot rule out the possibility that editing varies across sub-regions of cortex and striatum such that variations in precise dissection location could contribute to the observed variation. Interestingly, One subject had low rates of editing for all substrates in each brain region suggesting that this individual may harbor some trait locus that results in a global editing deficiency (although we cannot rule out a unique environmental insult). Regardless, the existence of this subject also provides compelling evidence for some upstream mechanism capable of affecting editing globally at all substrates across brain regions. Another subject harbored a deficiency in editing only at 5HT2C transcripts, which generalized across both brain regions. However, editing at each of the other sites was ‘normal’ compared to the rest of the cohort. We tested the hypothesis that a sequence polymorphism within the 5HT2C gene of this individual might affect a component of the secondary RNA structure required for A-to-I editing. However, analysis of the genomic sequence encoding hee predicted 5HT2C transcript RNA duplex did not reveal any polymorphisms. This implies that other factors or possibly polymorphisms at more remote locations could influence editing at this substrate in humans.

It is interesting to consider the prospect that the processes regulating editing are influenced by the environment and can be dynamically tuned throughout life providing adaptive plasticity. Alternatively, the differences could be relatively non-malleable and dictated by a molecular balance coded uniquely in each individual genome. In a further effort to discriminate these models, we looked at the patterns of editing in matched brain regions in 12 rhesus monkeys that where raised in a controlled laboratory environment. The variability in editing was generally much lower at each site in the monkey cohort compared to the humans suggesting that similar environmental conditions may foster the manifestation of similar editing profiles. Despite differences in variability, monkeys and humans displayed similar anatomical patterns of editing at some sites as highlighted in Fig. 1. The observation that these patterns are retained across primate species suggests that regulated expression of specific editing profiles in discrete brain regions confers some utility advantage and has been conserved through primate evolution.

The results of these studies indicate that there are likely global mechanisms responsible for regulating editing at all of these substrates typified by the case of global deficiency in A-to-I editing manifesting in each substrate and across both brain regions analyzed. Moreover, several examples of context specific regulation of editing were observed as some individuals demonstrated deficiencies in editing only at specific substrates and in specific brain regions. Taken together, these results imply that several distinct levels of regulation exist which can affect the efficiency of editing either globally or only in specific contexts. It is tempting to speculate that editing at different substrates is independently regulated by specific mechanisms inherent to each unique transcript. However, we cannot rule out contributions made by independent cell populations; the observed differences could result from altered regulation of editing in specific cells rather than at specific substrate transcripts.

To test the hypothesis that editing is effected by ADAR expression, we measured mRNA expression levels of ADAR1 and ADAR2 in each tissue sample and compared these levels to the extent of editing at each substrate. We observed a range of ADAR mRNA expression levels across tissue samples however none of the relative expression levels appeared to positively correlate with the extent of editing at any substrate analyzed in this study. Of note however, we did observe characteristic ratios of ADAR1:ADAR2 mRNA expression specific to striatum and cortex (Fig. 3). These fixed ratios may have important implications for the regulation of editing through potential competition between ADAR enzymes at certain editing sites.

It has been suggested by some studies that the 5HT2C editing could play a role in the etiology of the human genetic disorder PWS [20]. However, comparison of matched tissue samples from PWS patients and normal controls indicates similar editing patterns regardless of PWS diagnosis. The work presented here does not rule out the possibility that 5HT2C editing could be altered in PWS patients within certain discrete brain regions such as those regulating hunger and satiety. Unfortunately, attempts to procure precisely dissected brain tissue from ventromedial and lateral hypothalamus were unsuccessful due to the rarity of the disease and the limited availability of these relatively small brain structures.

This work clearly demonstrates that mechanisms for maintaining precise, context-specific editing profiles for certain edited transcripts are conserved across primate species. Divergent patterns observed in some human individuals suggest that while editing often falls within characteristic ranges, it can vary widely in certain circumstances. The fact that evolution has conserved editing within specific ranges across species suggests that the differences in editing observed in some human individuals from these studies are likely to have consequences for CNS function. It will be important to identify the neurological manifestations of altered editing profiles by studying larger human cohorts with detailed psychiatric and neurological histories. Identification of factors capable of influencing the editing profile will facilitate more complete understanding of feedback processes that influence the composition of edited transcripts in the brain.

## Materials and methods

### Tissue collection and processing

All human tissue was obtained from the NICHD Brain and Tissue Bank for Developmental Disorders (University of Maryland in Baltimore, MD) some of the samples were initially selected for a different study because they were obtained from individuals with Prader-Willi Syndrome. Covariant analysis indicated that Prader-Willi diagnosis did not significantly influence any of the parameters discussed here. Tissue was processed by crushing into fine powder while frozen using a ‘Cryo-cup’ grinder and pestle (Biospec products, Tulsa, OK) and mechanically homogenized in Tri-reagent (Ambion, Austin, TX). RNA was isolated from the Tri-reagent mixture according to manufacturer’s instructions and stored at−80C until further analysis. *Rhesus macaque* RNA samples were obtained as a gift from Karoly Mirnics (Vanderbilt University, Nashville, TN) and prepared as described in [21]. All monkeys were female Indian rhesus monkeys (*macaca mulatta*) from the colony at Oregon National Primate Research Center and were representatives of two groups divided by spontaneous physical activity as indicated in Mitchel et al. [19]. Covariant analysis did not indicate an effect of physical activity on editing at any of the substrates analyzed (Additional file 4: Figure S4).

### Tissue quality control

Choroid plexus is an ependymal tissue which expresses high levels of 5HT2C transcript bearing a unique editing profile. For this reason it was important to exclude any samples containing choroid plexus tissue from any 5HT2C editing analysis. This was accomplished by measuring the levels of clotting factor V (F5), a choroid plexus specific marker[22], in each tissue sample by Real-Time RT-PCR. Any samples containing detectable F5 were excluded from analysis of 5HT2C editing.

### Analysis of A-to-I editing

Editing profiles were determined by high-throughput multiplexed transcript (HTMTA) as described in [23] [12]. Reactions amplifying each respective substrate transcript were performed in parallel using one of 24 unique barcoded primer sets allowing for 24 sample multiplexing.

### Informatics

The FASTX-Toolkit was used to split the reads according each of the 24 different barcodes. For each read, we then used a script for pairwise alignments against all of the reference sequences allowing for only one mismatch (at the editing site) and assigned each match to its corresponding candidate gene. We then counted the nucleotide composition at the editing position and considered any adenosine at the corresponding site “not edited” and any guanosine “edited”, other nucleotides were detected at a very low rate presumably due to sequencing or polymerase errors and were not counted.

### Measuring mRNA expression

F5 was detected using a Fam labeled Taqman probe and primer set (Mm00484202_m1) and human ADAR expression was detected using probe and primer sets ADAR1 (Hs00241666_m1) and ADAR2 (Hs00953724_m1), and rhesus ADAR expression was detected with probes for ADAR1 (Hs00241666_m1) and ADAR2 (Rh00955199_m1). For qRT-PCR, first strand cDNA was synthesized using random primers using the High-Capacity Reverse Transcription Kit and expression was analyzed according to manufacturer’s instructions for Taqman real-time PCR (Applied Biosystems, Forster City, CA) on a CFX96 platform (Biorad, Hercules, CA). Parallel amplification efficiencies were verified by PCR miner algorithm [24] and data was analyzed by CFX manager (Biorad, Hercules, CA).

### Statistics

Analysis of covariance was performed in human samples by plotting the extent of editing at each respective site by Prader-Willi diagnosis, age, sex, and PMI. Covariance was determined by *T*-test for Prader-Willi diagnosis and sex; or linear regression for the continuous variables ADAR expression, age, and PMI. No significant effect was identified for any of these variables (*p* > .05). Standard statistical analysis (*t*-test) was used for pair wise comparisons. When analyzing correlations we used Pearson’s product moment to determine *p*-values.

## Additional files

Additional file 1: Figure S1. Plots for covariant analysis of various factors that were performed to determine the potential effects of sex, PWS diagnosis, age, and postmortem interval on the extent of editing at each respective site in the human cohort. **A** Comparing extent of editing at each site between males and females in both cortex and striatum does not reveal any significant effects on the extent of editing (*T*-test, *p* > .05 for each site). **B** Comparing the extent of editing at each site between patients diagnosed with PWS and and normal controls in both cortex and striatum does not reveal any significant effects on the extent of editing (*T*-test, *p* > .05 for each site). **C** Linear regression analysis comparing the age of each individual at the time of death does not reveal any significant effects on the extent of editing (*p* > .05). **D** Linear regression analysis comparing the post mortem interval to the extent of editing at each site does not reveal any significant effects on the extent of editing (*p* > .05). (DOCX 1894 kb)

Additional file 2: Figure S2. Plots for linear regression analysis for the effect of ADAR mRNA expression on the extent of editing at each site in humans. **A** Comparing the ADAR1 mRNA expression levels as determined by real-time PCR analysis to the extent of editing at each site does not reveal any significant effect on the extent of editing (*p* > .05). **B** Comparing the ADAR2 mRNA expression levels as determined by real-time PCR analysis to the extent of editing at each site does not reveal any significant effect on the extent of editing (*p* > .05). (DOCX 1356 kb)

Additional file 3: Figure S3. Plots for linear regression analysis for the effect of ADAR mRNA expression on the extent of editing at each site in monkeys. **A** Comparing the ADAR1 mRNA expression levels as determined by real-time PCR analysis to the extent of editing at each site does not reveal any significant effect on the extent of editing (*p* > .05). **B** Comparing the ADAR2 mRNA expression levels as determined by real-time PCR analysis to the extent of editing at each site does not reveal any significant effect on the extent of editing (*p* > .05). (DOCX 960 kb)

Additional file 4: Figure S4. Plots comparing the observed spontaneous activity of monkeys used in these studies to the extent of editing at each respective site. The two activity groups did not have significantly different extent of editing at any of the analyzed sites (*p* > .05). (DOCX 200 kb)
