# Supplementary material for: Comparative analysis of A-to-I editing in human and non-human primate brains reveals conserved patterns and context-dependent regulation of RNA editing
Source: Mol Brain. 2017 Apr 6;10:11. doi: 10.1186/s13041-017-0291-1 (PMC5382662; doi:10.1186/s13041-017-0291-1)
Supplement: Supplementary file 1 — Plots for covariant analysis of various factors that were performed to determine the potential effects of sex, PWS diagnosis, age, and postmortem interval on the extent of editing at each respective site in the human cohort. A Comparing extent of editing at each site between males and females in both cortex and striatum does not reveal any significant effects on the extent of editing (T-test, p > .05 for each site). B Comparing the extent of editing at each site between patients diagnosed with PWS and and normal controls in both cortex and striatum does not reveal any significant effects on the extent of editing (T-test, p > .05 for each site). C Linear regression analysis comparing the age of each individual at the time of death does not reveal any significant effects on the extent of editing (p > .05). D Linear regression analysis comparing the post mortem interval to the extent of editing at each site does not reveal any significant effects on the extent of editing (p > .05). (DOCX 1894 kb) [file 13041_2017_291_MOESM1_ESM.docx]

1. Editing by sex

1. Editing by PWS diagnosis

1. Linear regression of analysis of editing extent and age

Cortex

Striatum

1. Linear regression of editing and PMI

Cortex

Striatum
