# Supplementary material for: Comparative analysis of A-to-I editing in human and non-human primate brains reveals conserved patterns and context-dependent regulation of RNA editing
Source: Mol Brain. 2017 Apr 6;10:11. doi: 10.1186/s13041-017-0291-1 (PMC5382662; doi:10.1186/s13041-017-0291-1)
Supplement: Supplementary file 2 — Plots for linear regression analysis for the effect of ADAR mRNA expression on the extent of editing at each site in humans. A Comparing the ADAR1 mRNA expression levels as determined by real-time PCR analysis to the extent of editing at each site does not reveal any significant effect on the extent of editing (p > .05). B Comparing the ADAR2 mRNA expression levels as determined by real-time PCR analysis to the extent of editing at each site does not reveal any significant effect on the extent of editing (p > .05). (DOCX 1356 kb) [file 13041_2017_291_MOESM2_ESM.docx]

1. Linear regression of editing and ADAR1 RNA expression

Cortex

Striatum

1. Linear regression of editing and ADAR2 expression

Cortex

Striatum
