# Supplementary material for: Comparative analysis of A-to-I editing in human and non-human primate brains reveals conserved patterns and context-dependent regulation of RNA editing
Source: Mol Brain. 2017 Apr 6;10:11. doi: 10.1186/s13041-017-0291-1 (PMC5382662; doi:10.1186/s13041-017-0291-1)
Supplement: Supplementary file 4 — Plots comparing the observed spontaneous activity of monkeys used in these studies to the extent of editing at each respective site. The two activity groups did not have significantly different extent of editing at any of the analyzed sites (p > .05). (DOCX 200 kb) [file 13041_2017_291_MOESM4_ESM.docx]

Effect of exercise on editing profile in monkeys
